# From prediction to practice: artificial intelligence as an enabling layer in liver transplant care – reflections on ILTS 2025

**DOI:** 10.3389/frtra.2026.1879684

**Published:** 2026-06-10

**Authors:** Ali Zarrinpar, Avery F. Orinion

**Affiliations:** Department of Surgery, University of Florida, Gainesville, FL, United States

**Keywords:** artificial intelligence, clinical decision support, immunosuppression dosing, liver transplantation, risk stratification, translational medicine

## Abstract

Liver transplantation has become increasingly complex and data-intensive, demanding rapid, high-stakes clinical decisions as patient and organ conditions evolve. The surge of multidimensional clinical datasets has sparked strong interest in artificial intelligence (AI) and machine-learning (ML) as decision-support tools. The 2025 International Liver Transplantation Society (ILTS) annual meeting in Singapore highlighted that AI is now permeating every facet of liver transplantation, from donor-recipient matching and intra-operative navigation to post-transplant pharmacologic management. Early-stage models have shown impressive predictive performance in risk stratification for rejection, infection, and postoperative complications, as well as in individualized immunosuppression dosing and AI-assisted imaging/histopathology. However, real-world implementation has exposed persistent obstacles: data silos, limited generalizability, suboptimal explainability, and workflow friction that can erode clinician trust. Poor integration into existing clinical workflows may also increase cognitive burden for clinicians and reduce adoption of otherwise promising tools. This perspective synthesizes the ILTS 2025 messages and recent transplant literature, proposing a pragmatic three-stage roadmap for AI adoption over the next five years. The roadmap moves from near-term, well-validated decision-support alerts integrated into electronic health records, through mid-term hybrid human-AI teams that embed explainable AI (XAI) into clinical workflows, to long-term global AI consortia that foster federated learning and equitable model deployment. Central to this vision is a shift from “AI as an autonomous decision-maker” to “augmented intelligence that amplifies human expertise while remaining transparent and equitable,” ensuring that AI serves as an enabling layer that augments, rather than replaces, clinical judgment.

## Introduction

Liver transplantation epitomizes clinical complexity. The procedure hinges on a delicate balance of immune modulation, dynamic pharmacokinetics, and the equitable allocation of a scarce organ resource, not to mention the complexities of the operation and the management of critically ill patients with multiple medical problems. Traditional decision-making relies heavily on heuristics, mental shortcuts that simplify the integration of massive, heterogeneous data streams, but this approach can miss subtle patterns that influence outcomes (1). The program at the 2025 annual meeting of ILTS highlighted AI as a means to augment, not replace, human judgment by systematically extracting information from the “all data” environment that now surrounds every transplant center, including national registries, electronic health records, claims databases, and emerging wearable sensors.

**Table 1:** Current applications of AI in liver transplantation.

Application area	Example use	Potential benefit	Key limitation
Donor-recipient Matching	Allocation prediction models	Improved graft utilization	Limited generalizability
Immunosuppression Management	Dosing algorithms	Personalized therapy	Limited prospective validation
Imaging and Pathology	Steatosis quantification	Faster standardized assessment	Single-center training datasets
Surgical Planning	AR and 3-D segmentation	Improved operative precision	Workflow integration
ICU/Resource Allocation	Sepsis and acuity prediction	Earlier intervention	Alert fatigue
Post-Transplant Monitoring	Graft fibrosis prediction	Reduced surveillance burden	Retrospective study design

The talk titled *AI in Liver Transplantation: Choosing the Optimal Model for Your Center* by one of the authors (AZ) aimed to provide a roadmap for clinicians and administrators who are considering the adoption of AI tools. The following sections expand on those ideas, deepen the discussion of current advances, examine the practical limitations that surfaced at the meeting, and outline a forward-looking agenda that aligns with the ethical and operational imperatives of the transplant community.

## Current advances

### Clinical decision support and real-time prediction

AI/ML is already shaping several near-term domains: 1) advanced risk stratification for rejection, infection and postoperative complications (1); 2) individualized immunosuppression dosing and immune-response modeling (2); 3) AI-assisted pathology and radiology, e.g., deep-learning networks that quantify hepatic steatosis on whole-slide images and frozen donor biopsies (3–5), and automatic liver-segmentation algorithms that outperform semi-automatic methods (6); and 4) integration of predictive models into clinical workflows and EHR dashboards. These applications illustrate how AI can extract signals from multidimensional datasets that are beyond the reach of conventional analytic approaches.

### Augmented surgical visualization

The advent of real-time augmented reality (AR) overlays during hepatectomy and vascular anastomosis marks a concrete step toward intra-operative precision. By projecting three-dimensional reconstructions of donor vasculature onto the surgical field, surgeons can anticipate anatomical challenges before making an incision, potentially reducing intra-operative error rates and improving graft perfusion. The ILTS 2025 audience saw several case studies in which AR-guided navigation correlated with shorter warm-ischemia times and lower rates of vascular complications.

Beyond intra-operative guidance, AI-driven imaging is reshaping how surgeons acquire those 3-D models. Recent reviews of AI in liver-disease imaging demonstrate that deep-learning algorithms now automate fibrosis and steatosis quantification, improve lesion detection on ultrasound, CT and MRI, and consequently supply more reliable anatomical data for surgical planning. This enhanced imaging pipeline shortens the gap between pre-operative scans and intra-operative navigation, allowing surgeons to act on higher-quality, AI-validated maps of the liver parenchyma and tumor burden (7). This includes the generation of patient-specific virtual liver models. AI-generated virtual liver models derived from pre-operative imaging may further improve surgical planning and assessment of resection complexity (8). AI based 3-D segmentation tools are also improving liver volumetric analysis and intra-operative planning by providing faster and more standardized assessments of donor anatomy and residual liver volume (9). Together, AR-enhanced visualization, AI-augmented imaging, automated virtual-model generation, and high-fidelity 3-D segmentation constitute a convergent ecosystem of “augmented intelligence.” This ecosystem not only sharpens surgical precision but also standardizes pre-operative planning, reduces reliance on subjective interpretation, and creates a scalable foundation for equitable, data-driven liver transplantation.

### Operational optimization and resource allocation

AI is increasingly being integrated into transplant logistics and resource management, extending beyond bedside decision support to improve system-wide efficiency. By combining national registry data, operative schedules, and longitudinal clinical information, machine-learning models can forecast ICU and operating-room demands, optimize organ allocation, and predict graft viability, thereby reducing ischemia times and improving organ utilization.

Multiple studies have demonstrated the value of AI-driven risk stratification and allocation models in improving donor-recipient matching, perioperative planning, and predicting graft failure. For instance, Molinari et al. identified key predictors of 90-day mortality in more than 30,000 liver transplant recipients, supporting improved peri-operative triage and resource planning (10). Similarly, machine-learning allocation models developed by Lau et al. and Bertsimas et al. demonstrated improved prediction of graft failure and waitlist mortality compared with conventional approaches (11, 12). Longitudinal deep-learning models have also shown promise in post-transplant monitoring, enabling earlier identification of high-risk recipients and prediction of graft fibrosis using serial laboratory and clinical data (13, 14). Collectively, these studies highlight AI's ability to improve operational efficiency, organ utilization, and post-transplant care, although prospective validation and workflow integration continue to be ongoing challenges.

### Aligning patient acuity with resources

AI-driven acuity models are increasingly helping transplant programs match patient severity with available critical-care resources, including ICU beds, dialysis support, staffing, and operating-room access. By transforming high-dimensional clinical data into real-time risk estimates, these systems enable earlier intervention and more efficient allocation of limited resources.

Several studies have demonstrated that AI-based acuity models can improve peri-operative and ICU risk stratification by identifying patients at increased risk for complications such as acute kidney injury, sepsis, and hemodynamic instability (15–18). These systems may support earlier intervention, improve ICU resource allocation, and enhance operational planning through continuous analysis of physiological and laboratory data. Current applications of artificial intelligence in liver transplantation, along with their potential benefits and limitations, are summarized in Table 1.

Together, these approaches demonstrate how AI can improve alignment between patient acuity and resource intensity by anticipating ICU demand, identifying patients requiring specialized support, and enabling more adaptive clinical operations. Such systems may ultimately improve both efficiency and equity in liver transplantation care.

### Therapeutic innovation and drug discovery

Generative AI platforms are accelerating the design of novel therapeutic molecules and facilitating drug-repurposing efforts. The AI-Enabled Immunosuppression Management (AIIM) trial, due to be initiated soon, tests an augmented-intelligence dosing algorithm for tacrolimus with the explicit goal of preserving renal function while maintaining adequate graft protection. Early protocol outlines suggest that the trial will integrate continuous therapeutic drug monitoring data with pharmacogenomic inputs to generate patient-specific dosing recommendations (https://clinicaltrials.gov/study/NCT05432765). The AIIM trial is based partly on a complementary line of evidence from a phase-2, partially blinded, randomized study of phenotypic-personalized medicine (PPM)-guided tacrolimus dosing. In 62 liver-transplant recipients, the PPM group achieved a markedly lower proportion of post-transplant days with large (> 2 ng/mL) deviations from target trough levels (24.2% vs. 38.4% in the standard-of-care arm; *Δ* = -14.2%; *P* = 0.029) without an increase in adverse events. These results demonstrate that algorithmic, phenotype-driven dosing can tighten therapeutic windows and may reduce tacrolimus-related nephrotoxicity (19).

Together, the AIIM trial and the Khong et al. findings illustrate a broader shift: AI is moving from static risk scores toward dynamic, data-driven dosing platforms that continuously learn from each patient's biochemical and genomic profile. By automating complex pharmacokinetic modeling and suggesting individualized adjustments in real time, generative-AI approaches promise to improve graft survival, lessen drug-related complications, and streamline the workflow of transplant pharmacology.

## Limitations and practical barriers

Enthusiasm for AI in liver transplantation must be balanced against several practical limitations. Many published AI models are retrospective in design, developed using single-center or proprietary datasets and perform well during internal validation, but decline in accuracy when applied to external populations. Retrospective analyses are also vulnerable to selection bias, missing data, and limited prospective validation. This loss of performance may reflect overfitting, inconsistent variable definitions, heterogeneous data quality, and limited integration with existing electronic health record systems. As a result, AI tools that appear promising in development may offer only modest or uncertain benefit over traditional clinical scoring systems in real-world practice.

The UK Transplant Benefit Score (TBS) illustrates these risks. Introduced in 2018 to prioritize liver allocation according to predicted net survival benefit, the TBS was intended to reduce waitlist mortality and maximize life-years gained through a benefit-based allocation model (20). However, subsequent analyses showed that the model produced counterintuitive results for patients with chronic liver disease who developed hepatocellular carcinoma (HCC). Rather than increasing priority, the addition of HCC paradoxically lowered the likelihood of organ allocation by predicting improved survival without transplant (21). Lee et al. emphasized that this type of “black-box” decision-making can arise from spurious correlations in training data and that allocation systems must be transparent, interpretable, and continuously audited if they are to maintain clinician and public trust (15).

The TBS debate also highlights the difficulty of balancing urgency, utility, and equity. MELD-based systems in the United States and Eurotransplant regions have been criticized for potentially over-prioritizing HCC through exception points, yet these adjustments attempt to account for cancer-related mortality risk not captured by liver function scores alone (22). Conversely, defenders of the UK approach noted that TBS-based allocation reduced short-term waitlist mortality before the COVID-19 pandemic and reflected the unavoidable tradeoffs of organ scarcity (23). Together, these perspectives show that algorithmic optimization does not automatically translate into equitable allocation.

A second cautionary example comes from the UNOS cardiac-transplant database, where neural networks, random forests, and gradient-boosting models produced only modest improvements in 1-year survival prediction compared with standard logistic regression, with C-statistics no higher than 0.66 (24). This finding underscores that model sophistication cannot compensate for limitations in dataset quality, clinical heterogeneity, or missing variables.

Accordingly, AI tools in transplantation should be implemented through staged and transparent deployment: internal testing, external validation across diverse populations, prospective evaluation within clinical workflows, integration into EHR-based allocation and scheduling systems, and continuous monitoring for equity, calibration, and unintended disparities. The UK TBS experience demonstrates that even sophisticated models can worsen inequities when their assumptions are poorly understood or insufficiently tested. With rigorous oversight, however, AI-driven operational optimization may still improve the fairness, efficiency, and reliability of transplant care.

Together, these examples illustrate three core practical barriers:

1. Data provenance and generalizability- reliance on narrow, internally curated datasets limits external transportability.
2. Model interpretability and workflow fit- without explainable outputs and native EHR embedding, clinicians encounter alarm fatigue and mistrust.
3. Equity and outcome relevance- algorithms that are not rigorously vetted across diverse patient populations can inadvertently exacerbate existing disparities.

Addressing these issues will require multi-institutional data sharing, standardized variable ontologies, and iterative, prospective validation before AI tools become routine components of transplant decision-making.

Explainability emerged as another critical bottleneck. Clinicians are understandably hesitant to act on a black-box recommendation without insight into the underlying drivers. The concept of Explainable AI (XAI) was presented as a pathway to build trust, with platforms such as IBM's Watson XAI providing visualizations of feature importance and model uncertainty. Yet the practical implementation of XAI within the high-tempo environment of transplant surgery remains an open question, particularly when the urgency of decision-making leaves little time for deliberation over probabilistic outputs.

In addition to interpretability, patient safety and clinician workload must remain vital considerations during AI implementation. Excessive EHR-based alerts may contribute to alarm fatigue, potentially reducing responsiveness to clinical recommendations. Over reliance on outputs may also introduce bias, where clinicians defer to model recommendations despite conflicting clinical judgement. Accordingly, AI systems should function as supportive adjuncts rather than autonomous decision makers and must preserve clear pathways for both clinician override and accountability.

Regulatory and ethical considerations also loom large. Institutional review boards and national agencies often lack clear frameworks for evaluating AI-driven interventions, leading to delays in approval and, occasionally, outright rejection of promising tools. Moreover, the potential for algorithmic bias, stemming from skewed training data or hidden confounders, poses a risk of perpetuating existing inequities in organ allocation and postoperative care.

## Future directions

### Near-term (≤ 1 year)

The most immediate priority is the creation of standardized, multi-center data commons that pool heterogeneous transplant datasets while preserving patient privacy through federated learning techniques. By training models on a broader spectrum of practice patterns and patient demographics, we can improve generalizability and reduce over-fitting. Parallel to data consolidation, clinicians should demand that every AI module shipped into the EHR be accompanied by an XAI dashboard that surfaces the top contributing variables for each prediction, thereby offering a transparent rationale that clinicians can quickly evaluate.

Pilot clinical trials that examine AI-augmented decision support for immunosuppression dosing, such as the AIIM trial, should incorporate predefined safety thresholds and real-time monitoring of adverse events. These studies will provide the first high-quality evidence of whether augmented intelligence can meaningfully preserve renal function without compromising graft integrity.

### Mid-term (1–3 years)

As trust and technical infrastructure mature, transplant programs can transition from isolated decision-support alerts to hybrid human-AI teams. In this model, AI continuously generates risk stratifications and suggested interventions, while the transplant surgeon or hepatologist validates and contextualizes the recommendation. Such a collaborative workflow preserves ultimate clinical accountability while leveraging the pattern-recognition strength of algorithms.

Dynamic allocation algorithms, powered by reinforcement learning, can be iteratively refined using real-world outcomes, ensuring that donor-recipient matching evolves in response to shifting epidemiology and organ availability. Crucially, these algorithms must embed equity constraints, such as balance across geographic regions and disease severity, to avoid reproducing historical disparities.

### Long-term (∼ 5 years)

Envisioning the next half-decade, the transplant community should aim for a global AI consortium that federates data across continents, enabling models that are truly representative of diverse populations. This consortium would serve as a repository for continually updated, validated AI tools that any accredited center could deploy with minimal local adaptation.

Virtual clinical trials, wherein AI-simulated patient cohorts test novel therapeutic hypotheses before human enrolment, could compress the translational timeline for new immunosuppressants or organ-preservation solutions. By iterating on simulated data, researchers would identify the most promising candidates for expensive, resource-intensive human trials, thereby accelerating innovation while conserving funds.

## Discussion

The trajectory of AI in liver transplantation mirrors the early adoption curves of transformative technologies such as the internet and mobile telephony: initially widening the gap between early adopters and resource-limited sites, then gradually democratizing access as standards mature. The central insight from ILTS 2025 is that augmentation, not automation, should be the guiding principle. Augmented intelligence acknowledges the irreplaceable nuance of clinical expertise and frames AI as a “trusted partner” that surfaces hidden patterns, standardizes complex calculations, and reduces cognitive load.

From a philosophical standpoint, the distinction between *prediction* and *actionability* is critical. Many current models excel at forecasting a 30-day mortality probability but stop short of suggesting concrete management changes that improve that probability. Bridging this gap requires interdisciplinary collaboration among transplant surgeons, data scientists, ethicists, and health-system engineers to co-design interventions that are both scientifically sound and operationally feasible.

Equity remains the overarching ethical compass. The ILTS 2025 discussion warned that unchecked AI could inadvertently prioritize patients with more extensive data footprints, often those treated at high-volume academic centers, over those from underserved regions. By mandating that all AI development pipelines include bias audits, demographic subgroup performance reporting, and transparent fairness metrics, the transplant community can harness AI as an equalizer rather than a divider.

Finally, the concept of continuous learning systems, algorithms that update their parameters as new data flow in, offers a promising avenue to keep models current amidst evolving practice patterns, emerging viral threats, and shifting organ allocation policies. However, such systems must be paired with robust governance structures to prevent drift, preserve patient safety, and maintain regulatory compliance.

## Data Availability

The original contributions presented in the study are included in the article/Supplementary Material, further inquiries can be directed to the corresponding author.
